# Cervical dilatation patterns of ‘low‐risk’ women with spontaneous labour and normal perinatal outcomes: a systematic review

**DOI:** 10.1111/1471-0528.14930

**Published:** 2017-11-03

**Authors:** OT Oladapo, V Diaz, M Bonet, E Abalos, SS Thwin, H Souza, G Perdoná, JP Souza, AM Gülmezoglu

**Affiliations:** ^1^ UNDP/UNFPA/UNICEF/WHO/World Bank Special Programme of Research, Development and Research Training in Human Reproduction (HRP) Department of Reproductive Health and Research World Health Organization Geneva Switzerland; ^2^ Centro Rosarino de Estudios Perinatales Moreno Rosario Argentina; ^3^ Department of Social Medicine Ribeirão Preto Medical School University of São Paulo Ribeirão Preto Brazil

**Keywords:** Cervical dilatation, first‐stage labour, labour curve, labour dystocia, labour progression, spontaneous labour

## Abstract

**Background:**

The call for women‐centred approaches to reduce labour interventions, particularly primary caesarean section, has renewed an interest in gaining a better understanding of natural labour progression.

**Objective:**

To synthesise available data on the cervical dilatation patterns during spontaneous labour of ‘low‐risk’ women with normal perinatal outcomes.

**Search strategy:**

PubMed, EMBASE, CINAHL, POPLINE, Global Health Library, and reference lists of eligible studies.

**Selection criteria:**

Observational studies and other study designs.

**Data collection and analysis:**

Two authors extracted data on: maternal characteristics; labour interventions; the duration of labour centimetre by centimetre; and the duration of labour from dilatation at admission through to 10 cm. We pooled data across studies using weighted medians and employed the Bootstrap‐*t* method to generate the corresponding confidence bounds.

**Main results:**

Seven observational studies describing labour patterns for 99 971 women met our inclusion criteria. The median time to advance by 1 cm in nulliparous women was longer than 1 hour until a dilatation of 5 cm was reached, with markedly rapid progress after 6 cm. Similar labour progression patterns were observed in parous women. The 95th percentiles for both parity groups suggest that it was not uncommon for some women to reach 10 cm, despite dilatation rates that were much slower than the 1‐cm/hour threshold for most part of their first stage of labours.

**Conclusion:**

An expectation of a minimum cervical dilatation threshold of 1 cm/hour throughout the first stage of labour is unrealistic for most healthy nulliparous and parous women. Our findings call into question the universal application of clinical standards that are conceptually based on an expectation of linear labour progress in all women.

**Funding:**

UNDP/UNFPA/UNICEF/WHO/World Bank Special Programme of Research, Development and Research Training in Human Reproduction (HRP), Department of Reproductive Health and Research, World Health Organization, and the United States Agency for International Development (USAID).

**Tweetable abstract:**

Cervical dilatation threshold of 1 cm/hour throughout labour is unrealistic for most women, regardless of parity.

## Tweetable abstract

Cervical dilatation threshold of 1 cm/hour throughout labour is unrealistic for most women, regardless of parity.

## Introduction

The landmark studies of Emmanuel Friedman in the 1950s and 1960s on normal and abnormal labour progression have continued to influence labour management until today.^1^, ^2^, ^3^, ^4^, ^5^ Since the early 2000s, however, there is increasing evidence to suggest that the described relationship between cervical dilatation and duration of the first stage of labour, and the definitions of labour protraction and arrest that have informed obstetric practice for over six decades, may be inappropriate.^6^, ^7^ In practice, identifying abnormally progressing labour that justifies a medical intervention is often challenging. Thus, ‘failure of labour to progress’—a poorly defined but generally accepted term—has become a leading indication for oxytocin augmentation and primary caesarean section (CS). The need to medically expedite birth on the grounds of slow labour accounts for the rapid escalation in global rates of labour augmentation and CS in the last two decades.^8^ This interventionist approach, including the ‘active management of labour’ model of care, is likely to interfere with a woman's intrinsic capacity to give birth and to negatively impact on her birth experience and health outcomes.^9^

The call for women‐centred approaches to reduce labour and childbirth interventions, particularly primary CS, has renewed interest in a better understanding of natural labour progression.^10^, ^11^ This has become more critical because of the variations in current labour practices and the characteristics of pregnant women compared with when Friedman conducted his studies.^12^ An important derivative of Friedman's work—the ‘1‐cm/hour’ alert line of the partograph—has come under intense scrutiny as a result of studies suggesting that labour can indeed be slower than the limits earlier proposed.^6^, ^7^, ^13^ Studies published in the last decade on ‘natural’ labour progression have prompted a few international organisations to revise their labour management guidelines to accommodate a cervical dilatation rate slower than 1 cm/hour as the normal threshold.^11^, ^14^ In spite of these developments, several obstetric textbooks and international guidelines still maintain 1 cm/hour as the minimum dilatation threshold that should be expected in all women.^15^, ^16^ This lack of international consensus calls for a systematic evaluation of the available studies on this topic to justify a review of global guidance on assessment of labour progression. The aim of this review was to synthesise available data on cervical dilatation patterns during the spontaneous labour of ‘low‐risk’ women with normal perinatal outcomes.

## Methods

We conducted this review in accordance with the Preferred Reporting Items for Systematic Reviews and Meta‐Analyses (PRISMA) guidelines, and followed a protocol (PROSPERO2016:CRD42016053892).^17^ Eligible studies included published and unpublished observational studies reporting cervical dilatation over time for low‐risk women with spontaneous labour and normal perinatal outcomes. We considered but did not identify randomised and non‐randomised trials in which observations of cervical dilatation patterns were reported for our population of interest. We included studies where the study population was defined as women ‘without risk factors for complications’ or women who were deemed to be ‘low‐risk’, or with clearly defined criteria including at the minimum a singleton pregnancy, near‐term or term pregnancy, and cephalic (or vertex) presentation, and a labour progression that ended in a vaginal birth or reached full cervical dilatation. ‘Normal perinatal outcomes’ were as defined by the primary study authors, but must include birth of a live baby with an Apgar score of ≥7 at 5 minutes. We excluded studies that estimated the linear cervical dilatation rate from the total duration of labour without describing the labour progression patterns throughout the first stage, and those that applied an ‘active management of labour’ protocol in the management of all study participants.

We searched PubMed, EMBASE, CINAHL, POPLINE, Global Health Library, and the reference lists of eligible studies, and contacted authors for any further published and unpublished work. No date or language restrictions were applied. The detailed search strategies are included in Appendix S1. Two review authors independently performed initial screening of search outputs, identified eligible studies, and extracted the data. A third author verified eligible studies and checked data for errors. Any discrepancies were resolved through discussion.

Outcomes of interest consisted of baseline information (including demographic and reproductive characteristics and labour admission findings) and interventions during labour. To determine cervical dilatation patterns, we extracted data on the time (in hours) needed to gain 1 cm as labour progresses through to 10 cm (i.e. the ‘traverse time’ or ‘sojourn time’ from 2 to 3 cm, 3 to 4 cm, etc.) To assess potential differences in labour patterns based on cervical dilatation on admission, we extracted data on the cumulative labour duration from the cervical dilatation on admission (i.e. at 2, 3, 4, 5, and 6 cm) at an interval of 1 cm until 10 cm. All time‐related variables were extracted using the reported measure of central tendency (median or mean) and their corresponding distribution. Data were extracted according to two parity groups: nulliparous (parity = 0) and parous (parity ≥ 1) women.

We synthesised data on cervical dilatation patterns to generate aggregate estimates of time to gain 1 cm from one level of cervical dilatation to the other (i.e. centimetre by centimetre), and time to advance by 1 cm from cervical dilatation at admission, until 10 cm. For studies reporting medians, we determined the weighted median of time (in hours) to pool data across studies. The weighted approach, based on the number of women providing the data from each study, took into consideration the individual study size. To derive confidence bounds for pooled medians for the centimetre by centimetre labour duration, we combined samples generated with quantiles and sample sizes assuming uniform probability, and used a two‐level Bootstrap‐*t* method to estimate standard errors and percentiles of the Student's *t*‐statistic to compute confidence intervals.^18^ We used sas 9.4 (SAS Institute Inc., Cary, NC, USA) and r‐cran 3.4.0 for these analyses.^19^

We designed a checklist and criteria to assess the methodological quality of the included studies to investigate their internal and external validity based on the following attributes: primary intent of the research question (evaluation of labour progression clearly described as primary study objective versus secondary analysis); representativeness of the study population (truly representative of low‐risk women, with a detailed description of normal perinatal outcomes, versus unclear representativeness because of a lack of details on the excluded risk factors and/or inadequate description of perinatal outcomes); ascertainment and temporality of the observations (prospective direct observations versus retrospective review of medical charts); adequacy of data points for valid assessment of cervical dilatation patterns for study participants (restriction of analysis to women with at least three versus two or fewer/unreported data points); and the use of a valid and robust approach for analysing labour progression and constructing the labour curve (advanced statistical and/or computational method versus graphical or other methods). The studies were assessed to be at low, moderate, or high risk of bias based on compliance with more than three, three, and fewer than three of the above criteria, respectively.

## Results

**Figure 1:**
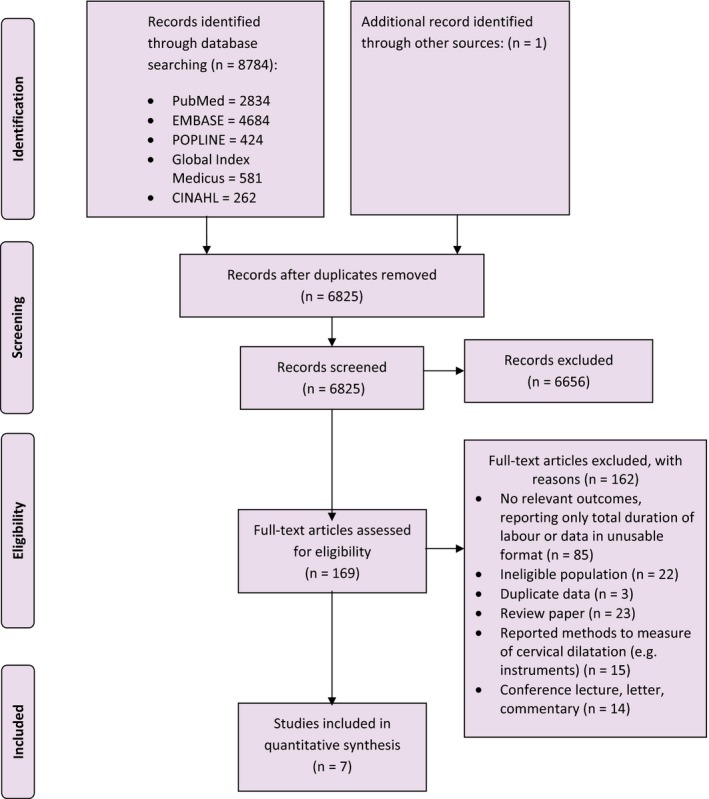
Detailed study selection process.

We prepared the results according to the proposed reporting checklist for meta‐analysis of observational studies.^20^ Of the 8785 citations obtained from the search strategies, 169 potentially eligible studies were identified for full‐text assessment (Figure 1). Seven observational studies conducted in the USA,^6^, ^7^, ^21^ China,^22^, ^23^ Japan,^24^ Nigeria (OT Oladapo et al., unpubl. data), and Uganda (OT Oladapo et al., unpubl. data) met our inclusion criteria.

Tables S1 and S2 show the characteristics of the included studies and corresponding study populations. Study populations were generally made up of the nationality where each study was conducted, except for studies in the USA.^6^, ^7^, ^21^ All of the studies provided data for nulliparous women (*n* = 43 148), whereas three studies provided data for parous women (*n* = 56 823) (OT Oladapo et al., unpubl. data).^6^, ^21^ Nulliparous women had mean ages of 20.3–28.0 years and gestational ages at delivery of 39.3–39.8 weeks. Parous women had mean ages of 22.7–30.9 years and gestational ages at delivery of 39.1–39.8 weeks.

Table 1 shows the baseline characteristics of women at labour admission, and the interventions received during labour. For nulliparous women the median cervical dilatation was 3.0–4.0 cm, with variable degrees of effacement, and for parous women the median cervical dilatation was 3.5–5.0 cm, with a considerable proportion of women with a well‐effaced cervix. Oxytocin augmentation varied from 0% (in a Chinese study)^23^ to 50% in a US study,^7^ for nulliparous women, and between 12^6^ and 45%^21^ in two US study populations for parous women. Epidural analgesia use was restricted to the US studies for both parity groups.^6^, ^7^, ^21^

**Table 1:** Baseline characteristics at admission and labour interventions in the study populations

Study	Year of publication	Ruptured membranes (%)	Effacement (median)	Effacement [p10 (%)]	Effacement [p90 (%)]	Cervical dilatation (median, cm)	Cervical dilatation (p10)	Cervical dilatation (p90)	Fetal station (median)	Fetal station (p10)	Fetal station (p90)	Vaginal exam/woman (median)	Vaginal exam/woman (p10)	Vaginal exam/woman (p90)	Amniotomy (%)	Oxytocin augmentation (%)	CS (%)	IVB (%)	Epidural (%)
**Nulliparous women (parity = 0)**																			
Chen et al.^23^	1986	—	—	—	—	3.6^c^			+0.4^c^	—	—	—	—	—	0.0	0.0	0.0	0.0	0.0
Zhang et al.^7^	2002	35.0	100% effaced in 38%	—	—	3.5	1.5	5.0	—	—	—	6	4	10	—	50.0	0.0	13.0	48.0
Suzuki et al.^24^	2010	—	—	—	—	2.9^c^	—	1.6^d^	—	—	—	4	—	—	—	6.5	0.0	0.0	0.0
Zhang et al.^6^	2010	29.0	85%	50	100	3	1	6	0	−2	1	6	3	11	—	20.0	0.2^e^	73.0	4.0
Zhang et al.^21^	2010	—	90%	60	100	4	1	7	−1	−3	0	5	1	9	—	47.0	0	12.0	84.0
Shi et al.^22^	2016	22.7	57% admitted at <3 cm	—	—	—	—	—	—	—	—	4	3	6	49.8	9.2	0.0	1.3	—
OT Oladapo et al. (unpubl. data)	2017	24.7	80% effaced in 42.4%	—	—	4	2	6	≤−1 in 73.5%	—	—	3	2	5	—	23.5	0.0	2.9	0.0
**Parous women (parity ≥ 1)**																			
Zhang et al.^a^, ^6^	2010	26	80%	45	100	3.5	2	7	0	−2	1	5	2	9	—	12.0	0.02^e^	45.0	5.5
Zhang et al.^b^, ^6^	2010	27	75%	30	100	3.5	1.5	6.5	−1	−3	1	5	2	9	—	12.0	0.05^e^	24.0	4.0
Zhang et al.^a^, ^21^	2010	—	90%	50	100	4.5	2	8	−1	−3	0	4	1	9	—	45.0	0.0	3.0	77.0
Zhang et al.^b^, ^21^	2010	—	80%	50	100	5	2	8	−2	−3	0	4	1	7	—	45.0	0.0	2.0	71.0
OT Oladapo et al. (unpubl. data)^a^	2017	19.8	80% effaced in 49.5%	—	—	4	2	6	≤−1 (in 71.0%)	—	—	3	2	4	—	29.8	0.0	1.3	0.0
OT Oladapo et al. (unpubl. data)^b^	2017	21.5	80% effaced in 47.2%	—	—	4	2	6	≤−1 (in 70.8%)	—	—	3	2	4	—	26.7	0.0	0.5	0.0

IVB, instrumental vaginal birth.

a Data only for parity = 1.

b Data only for parity > 1.

c Mean.

d SD.

e Second‐stage caesarean section (CS).

–, no data reported.

Tables S3 and S4, and Figure S1, present the risk of bias for the included studies. Of the seven studies examining nulliparous women, four were considered to be at low risk of bias (OT Oladapo et al., unpubl. data),^6^, ^21^, ^22^ two at moderate risk of bias,^7^, ^24^ and one at high risk of bias.^23^ All three studies examining parous women were assessed to be at low risk of bias (OT Oladapo et al., unpubl. data).^6^, ^21^

### Cervical dilatation patterns

#### Time to advance by 1 cm during the first stage of labour in nulliparous women

Table 2 shows the time required to advance from one level of cervical dilatation to the next. From six studies, the pooled median time to advance by 1 cm in nulliparous women was longer than 1 hour until a dilatation of 5 cm was reached (i.e. when the median dilatation rate became 1.09 cm/hour) (OT Oladapo et al., unpubl. data).^6^, ^7^, ^21^, ^22^, ^24^ The transition to more rapid progress started between 5 and 6 cm, but it was only after 6 cm that the dilatation rate doubled (Figure S2). The 95th percentiles of the time reported by individual studies suggest that it was not uncommon for women to spend more than 4 hours progressing from 3 to 4 cm, as well as from 4 to 5 cm. As labour progressed, the 95th percentiles show wide variability around the median for each level of cervical dilatation in each study; however, there is a considerable overlap in the distributions of women whose labours were slower than the median for their populations, but who achieved full dilatation when considered centimetre by centimetre (Figure S3). The exception is Suzuki et al.,^24^ who reported an even slower labour than all other studies. Based on the lowest values of 95th percentile data across studies, there were always women whose rates of dilatation never reached the 1‐cm/hour threshold except between 9 and 10 cm. The data show that it was not uncommon for women to achieve full cervical dilatation despite progressing at rates slower than the 1‐cm/hour threshold for the most part of their labours. The only study that reported the mean time to advance by 1 cm found similar patterns as were found in the studies reporting medians.^23^

**Table 2:** Time interval (in hours) at each stage of cervical dilatation for nulliparous women

Study	*n*	2–3 cm			3–4 cm			4–5 cm			5–6 cm			6–7 cm			7–8 cm			8–9 cm			9–10 cm		
		m	5th	95th	m	5th	95th	m	5th	95th	m	5th	95th	m	5th	95th	m	5th	95th	m	5th	95th	m	5th	95th
Zhang et al.^7^	1162	3.2	0.6	15.0	2.7	0.6	10.1	1.7	0.4	6.6	0.8	0.2	3.1	0.6	0.2	2.2	0.5	0.1	1.5	0.4	0.1	1.3	0.4	0.1	1.4
Suzuki et al.^24^	2369	7.5	2.7	21.0	6.2	2.2	17.7	4.8	1.5	15.7	3.3	1.0	10.7	2.6	0.7	9.3	1.8	0.5	6.8	1.0	0.2	4.4	0.9	0.3	2.6
Zhang et al.^6^	8690	—	—	—	1.20	—	6.60	0.90	—	4.50	0.60	—	2.60	0.5	—	1.80	0.40	—	1.40	0.40	—	1.30	0.40	—	1.20
Zhang et al.^21^	27 170	—	—	—	1.8	—	8.1	1.30	—	6.40	0.80	—	3.20	0.60	—	2.20	0.50	—	1.60	0.50	—	1.80	0.50	—	1.80
Shi et al.^22^	1091	2.67	—	7.20	2.00	—	4.20	1.71	—	4.00	1.00	—	2.50	1.00	—	2.30	0.90	—	2.10	1.00	—	2.50	0.33	—	1.00
OT Oladapo et al. (unpubl. data)	2166	—	—	—	2.82	0.60	13.33	1.72	0.38	7.83	1.19	0.23	6.17	0.66	0.09	4.92	0.25	0.02	3.10	—	—	—	—	—	—
Pooled values	42 648	5.28			2.00			1.46			0.92			0.70			0.55			0.52			0.49		
Lower confidence bound		5.07			1.89			1.39			0.89			0.68			0.53			0.50			0.48		
Upper confidence bound		5.46			2.11			1.52			0.96			0.73			0.57			0.53			0.51		
Median rate of dilatation (cm/hour)		0.19			0.50			0.68			1.09			1.43			1.82			1.92			2.04		
	***n***	m*	**1SD**	**2SD**	m*	**1SD**	**2SD**	m*	**1SD**	**2SD**	m*	**1SD**	**2SD**	m*	**1SD**	**2SD**	m*	**1SD**	**2SD**	m*	**1SD**	**2SD**	m*	**1SD**	**2SD**
Chen et al.^23^	500	—	—	—	1.39	1.15	2.30	1.03	0.99	1.98	0.81	0.97	1.94	0.54	0.60	1.20	0.46	0.41	0.82	0.50	0.44	0.88	0.56	0.49	0.98
Mean rate of dilatation (cm/hour)		—	—	—	0.72			0.97			1.23			1.85			2.17			2.00			1.79		

—, no data reported; 5th, 5th percentile; 95th, 95th percentile; m, median; m*, mean; SD, standard deviation.

#### Time to advance by 1 cm during the first stage of labour in parous women

Similar to nulliparous women, Table 3 shows that the pooled median time it took parous women to advance by 1 cm was longer than 1 hour until a cervical dilatation of 5 cm was reached (when the median dilatation rate became 1.49 cm/hour); however, the dilatation rate increased sharply and almost doubled between 5 and 6 cm, and then rose rapidly as it advanced towards 10 cm (Figure S2). As labour progressed, the 95th percentile data show considerable overlap in the distributions of women with labours that progressed more slowly than the medians and yet achieved full dilatation (Figure S4). When advancing from 4 to 5 cm, some women took between 3.30 and 8.05 hours, and when advancing from 5 to 6 cm, some women took between 1.60 and 6.24 hours. Based on the lowest values of 95th percentile data across studies, there were always women whose rates of dilatation never reached the 1‐cm/hour threshold until 7 cm.

**Table 3:** Time interval (in hours) at each stage of cervical dilatation, for parous women

Study	*n*	3–4 cm			4–5 cm			5–6 cm			6–7 cm			7–8 cm			8–9 cm			9–10 cm		
		m	5th	95th	m	5th	95th	m	5th	95th	m	5th	95th	m	5th	95th	m	5th	95th	m	5th	95th
Zhang et al.^a^, ^6^	6373	—	—	—	0.70	—	3.30	0.40	—	1.60	0.4	—	1.20	0.30	—	0.80	0.30	—	0.70	0.20	—	0.50
Zhang et al.^a^, ^21^	17 850	—	—	—	1.40	—	7.30	0.80	—	3.40	0.50	—	1.90	0.40	—	1.30	0.30	—	1.00	0.30	—	0.90
OT Oladapo et al. (unpubl. data)^a^	1488	2.42	0.41	14.18	1.37	0.25	7.65	0.79	0.13	4.95	0.33	0.03	3.67	0.09	0.00	2.69	—	—	—	—	—	—
Zhang et al.^b^, ^6^	11 765	—	—	—	0.70	—	3.50	0.40	—	1.60	0.3	—	1.20	0.30	—	0.70	0.20	—	0.60	0.20	—	0.50
Zhang et al.^b^, ^21^	17 395	—	—	—	1.40	—	7.00	0.80	—	3.40	0.50	—	1.80	0.40	—	1.20	0.30	—	0.90	0.30	—	0.80
OT Oladapo et al. (unpubl. data)^b^	1952	2.35	0.31	17.85	1.18	0.17	8.05	0.79	0.10	6.24	0.31	0.03	3.29	0.17	0.01	2.44	—	—	—	—	—	—
Pooled values	56 823	2.38			1.17			0.67			0.44			0.35			0.28			0.27		
Lower confidence bounds		1.41			1.15			0.66			0.43			0.34			0.27			0.26		
Upper confidence bounds		2.99			1.18			0.67			0.44			0.35			0.28			0.27		
Median rate of dilatation (cm/hour)		0.42			0.85			1.49			2.27			2.86			3.57			3.70		

—, no data reported; 5th, 5th percentile; 95th, 95th percentile; m, median.

a Data only for parity = 1.

b Data only for parity > 1.

#### Cumulative labour duration from cervical dilatation at admission in nulliparous women

Three studies reporting the median cumulative duration of labour from the dilatation at admission to the next centimetre, until 10 cm was reached, show the patterns reflected in the centimetre‐by‐centimetre progression (Table S5) (OT Oladapo et al., unpubl. data).^6^, ^21^ When estimated linearly, the total median time from any cervical dilatation at admission until 10 cm reflects a dilatation rate of less than 1 cm/hour: 2–10 cm (7.85 hours), 3–10 cm (6.44 hours), 4–10 cm (4.86 hours), 5–10 cm (3.44 hours), and 6–10 cm (2.86 hours). The rates became much faster as the dilatation at admission increased, similar to the observations in the centimetre‐by‐centimetre data; however, the corresponding 95th percentile data show a wide variability above the median duration. Figure S5 illustrates these 95th percentiles plotted as connected staircase lines for specific dilatation observed at admission. For example, in nulliparous women admitted with a dilatation of 2 cm, the pooled median time to reach 4 cm was 2.96 hours, but the 95th percentiles ranged between 11.20 and 12.60 hours. Likewise, the pooled median time to achieve a dilatation of 10 cm for women admitted at 4 cm was 4.86 hours, but the 95th percentiles were between 14.10 and 16.40 hours.

#### Cumulative labour duration from cervical dilatation at admission in parous women

One study reported data on the cumulative duration of labour based on the dilatation at admission for parous women (Table S6) (OT Oladapo et al., unpubl. data). The observed patterns are similar to those for nulliparous women. Figure S6 illustrates the 95th percentile data plotted as connected staircase lines for dilatation at admission. For example, in women admitted at 3 cm, the pooled median time to reach 5 cm was 3.49 hours, but the 95th percentiles ranged between 18.55 and 20.75 hours, and for women admitted at 4 cm, the pooled median time to reach 10 cm was 3.23 hours, but the 95th percentile ranged from 12.96 to 13.02 hours.

## Discussion

### Main findings

Our review shows that cervical dilatation patterns for low‐risk women are not linear. The overall labour progression pattern deviates considerably from the classic Friedman's curve that has been central to labour practice for several decades.^1^, ^2^, ^3^ In the early part of the period that is traditionally described as the active phase, we found that the rate of cervical dilatation may be slower, and in advanced labour the rate may indeed be faster than generally thought. Labour tends to become accelerative (i.e. >1 cm/hour) between 5 and 6 cm in both nulliparous and parous women. Thereafter, it escalates rapidly as labour becomes more advanced. An important finding across included studies is the wide variability in the distribution of cervical dilatation profiles, as shown by the 5th and 95th percentiles. Whereas the median cumulative duration of labour from cervical dilatation at admission through full dilatation is similar to the observations in earlier studies,^1^, ^2^, ^3^, ^25^, ^26^ the corresponding 95th percentiles across studies show that it is not uncommon for some women to experience much longer labour and yet give birth vaginally without adverse perinatal outcomes. Women admitted before 4 cm tend to progress very slowly, and could take close to 24 hours before achieving full cervical dilatation.

### Strength and limitations

To our knowledge, this is the first systematic review that has analysed cervical dilatation patterns centimetre by centimetre for the assessment of labour progression. We examined the cervical dilatation pattern from one level to the next, rather than estimate the average dilatation rate based on centimetres covered over a period of time. We included studies that have taken advantage of statistical advancements to minimise the challenges of studying labour progression.^27^ We minimised potential bias in the review process by searching major databases without any restrictions, and considered studies conducted as long ago as the 1950s. Our review included studies describing the cervical dilatation patterns for close to 100 000 women of diverse ethnicities, spread across major geographic regions of the world. We employed a pragmatic statistical approach to address the challenges of generating confidence bounds for pooled medians when only quantiles and sample sizes are provided, and other distributional parameters for the underlying study populations are not available. Nonetheless, a few limitations need to be highlighted.

The main limitations of this review relate to the inherent limitations in the design and conduct of the primary studies. Notable is the potential selection bias that could arise from the populations selected for individual studies (e.g. the exclusion of women who had CS during first stage, and the inclusion of women with variable use of oxytocin augmentation, epidural analgesia, and instrumental vaginal birth). Although it is possible that the synthesis of these studies could accentuate such bias, the general consistency in the observed patterns of cervical dilatation across all studies is reassuring. We believe that the inclusion of women with certain interventions, albeit variable across studies, strengthens the generalisability of our results to current obstetric practice, as the presented data reflect the diversity in healthcare practices within and across settings. Another limitation arises from the non‐uniformity in weights used for pooling medians. As women in the studies were not admitted at the same cervical dilatation, and thus would not have contributed data equally to every centimetre of cervical dilatation for their cohort, the use of the same sample size for weighting could have impacted the pooled values, especially for those reported for the early phase of labour (e.g. 2–3 cm and 3–4 cm). Therefore, aggregated values for the period traditionally described as the latent phase should be interpreted with caution.

### Interpretation

Our findings support the observations from other primary studies showing that the dilatation rate in healthy pregnant women could be slower than 1 cm/hour.^25^, ^26^, ^28^ Although such studies have generally selected women who received no obstetric intervention to explore the natural history of labour, in our review we found that women could have slower rates of cervical dilatation, even in contexts where obstetric interventions are the norm.

A remarkable finding of this review is the demonstration of nonlinear changes in cervical dilatation rates throughout the period commonly described as the active phase. For instance, up to 50% of the nulliparous population analysed progressed from a much slower (0.50 cm/hour at 3–4 cm) to a much faster (2.04 cm/hour at 9–10 cm) rate, compared with the accepted minimum threshold. The variability shown by the 95th percentiles across studies confirms that although it was not uncommon for some women to experience an even slower cervical dilatation rate as they progressed in labour, the nonlinear pattern remained preserved. Based on this finding, it may be more clinically beneficial to conceptualise labour pattern as a hyperbolic curve that is slower at the beginning of the traditional active phase and faster towards full dilatation, when prospectively making a clinical decision about labour progress. Although this may be difficult to implement in practice, especially because dilatation rates can change very quickly, even in the same woman, it has the prospect of reducing the premature diagnosis of dystocia in early labour, and could potentially reduce the unnecessary use of interventions to accelerate labour.

The clinical decision about whether or not interventions should be applied to accelerate labour requires an understanding of when a woman is truly in her phase of natural acceleration. Although the commonly agreed point of onset of accelerative labour is 4 cm, as popularised by the start of the partograph alert line, our review suggests that labour may indeed not begin to accelerate substantially until 6 cm in a considerable proportion of women. This observation from one of the studies in this review was used as the basis for the recent American College of Obstetricians and Gynaecologists’ (ACOG) recommendation that standards of care for the active phase of labour should only be applied when women have reached a 6‐cm threshold.^6^, ^11^ The patterns observed in the individual studies included in our review and the aggregated estimates provide even more robust evidence in support of this recommendation. We propose that the revision of norms and standards for labour care should evaluate the impact of these findings on the starting point of the partograph alert line.

The use of arbitrary statistical limits to define the boundaries for normal labour progression deserves a special mention. Since the pioneer studies of Friedman, the 95th percentiles have been arbitrarily taken as the upper limit of normal labour duration or dilatation rate. Although this offers the clinician a benchmark against which to evaluate women in labour, it does not imply that labour within this boundary cannot result in adverse outcomes. Rather, the 95th percentiles provide boundaries within which, when maternal and fetal conditions are reassuring, a woman should continue to be offered expectant, supportive, and woman‐centred labour care. This assertion is supported by the similarity between the labour progression patterns described in this review and those reported from study populations with risk factors and childbirth‐related morbidities.^29^, ^30^

## Conclusions

The opportunity to confidently assess natural labour progression without any intervention or interference in current obstetric practice is limited; however, new computational methods in the included studies offer an insight into what may have been responsible for the increasing medicalisation of birth over the last two decades. The overall labour progression data within and across studies show that an expectation of a minimum cervical dilatation threshold of 1 cm/hour throughout labour is unrealistic for most healthy nulliparous and parous women. To improve birth outcomes, it is essential to lower this expectation and conceptualise labour progress as a potentially hyperbolic rather than a linear process. The findings of our review call into question the application of standards that are currently in use based on research conducted more than 50 years ago. A potential direction for future research could be the use of individual‐level data set meta‐analysis, where statistical methodology using LMS (*Lambda, Mu, and Sigma*) parameters could be explored to construct normalised centile standards, similar to its application for the development of growth charts.^31^

### Disclosure of interests

Full disclosure of interests available to view online as supporting information.

### Contribution to authorship

OTO conceived the review and drafted the protocol, with input from MB, EA, JPS, and AMG. OTO worked with the WHO information specialists to build the search strategies and undertake the searches. VD and MB performed the initial screening of search outputs, identified eligible studies and extracted data. OTO verified eligible studies and checked data for errors. SST, HC, and GP performed all statistical analyses in the review. OTO drafted the manuscript. All authors interpreted the data and revised the data for intellectual content, and approved the article for publication.

### Details of ethics approval

No ethical approval was required for this study.

### Funding

The UNDP/UNFPA/UNICEF/WHO/World Bank Special Programme of Research, Development and Research Training in Human Reproduction (HRP), Department of Reproductive Health and Research, World Health Organization funded the preparation of this systematic review through a grant from the United States Agency for International Development (USAID), as part of the evidence base preparation towards the WHO recommendations on intrapartum care for a positive childbirth experience.

## Supporting information


**Figure S1.** Risk of bias assessment.

**Figure S2.** Cervical dilatation patterns according to pooled median times to advance centimetre by centimetre.

**Figure S3.** Panel showing the distribution of median time to gain 1 cm in nulliparous women, by study.

**Figure S4.** Panel showing the distribution of time to gain 1 cm in parous women, by study.

**Figure S5.** Panel showing 95th percentiles of cumulative duration of labour from admission, by study among nulliparous women.

**Figure S6.** Panel showing 95th percentiles of cumulative duration of labour from admission, by study, among parous women.

**Table S1.** Characteristics of included studies and study populations (nulliparous).

**Table S2.** Characteristics of included studies and study populations (parous).

**Table S3.** Risk of bias assessment of included studies.

**Table S4.** Overall risk of bias assessment.

**Table S5.** Cumulative duration of labour (in hours) from cervical dilatation at admission in nulliparous women.

**Table S6.** Cumulative duration of labour (in hours) from cervical dilatation at admission in parous women (parity = 1 or >1).

**Appendix S1.** Search strategies (date of search: 15 December 2016).
