# Supplementary figures and images for: Cervical dilatation patterns of ‘low‐risk’ women with spontaneous labour and normal perinatal outcomes: a systematic review
Source: BJOG. 2017 Nov 3;125(8):944–54. doi: 10.1111/1471-0528.14930 (PMC6033146; doi:10.1111/1471-0528.14930)

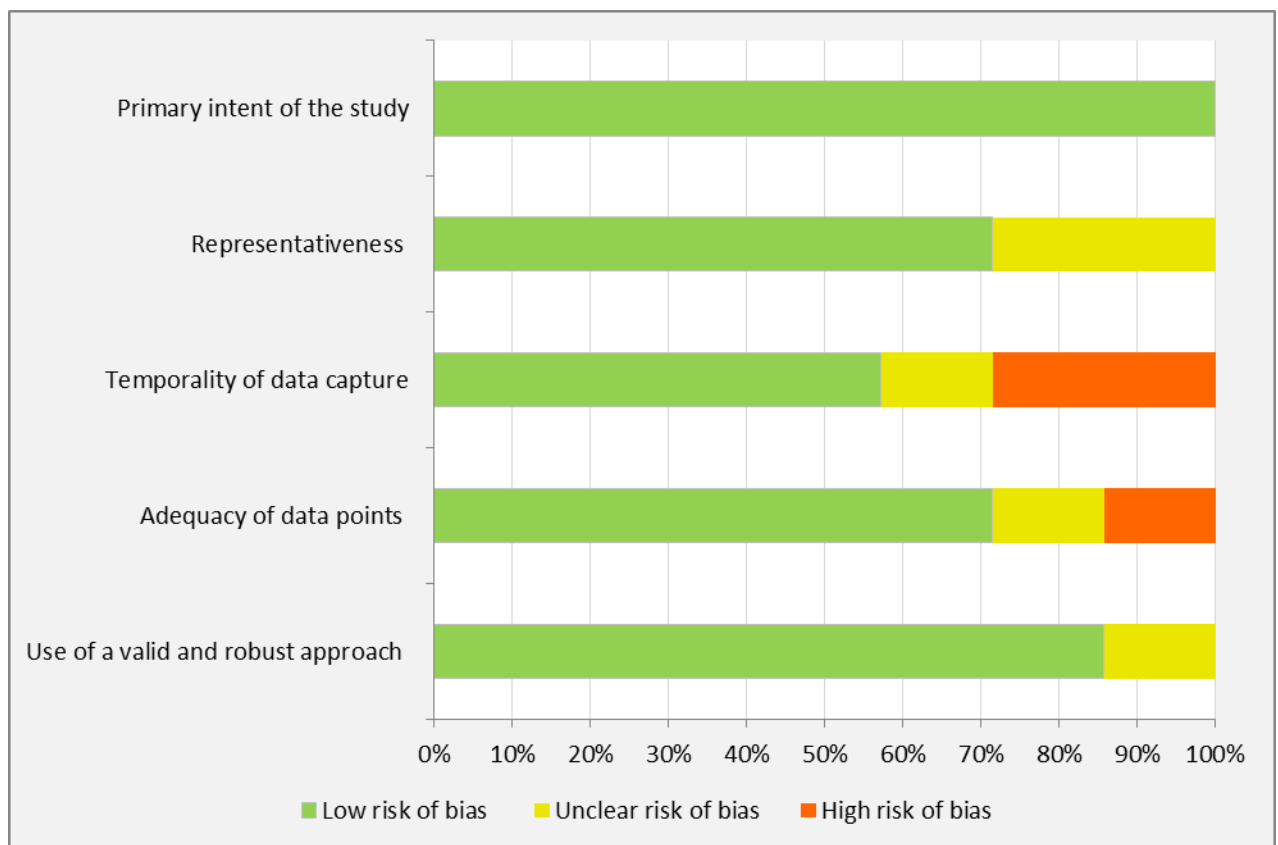

**Figure S1.** Risk of bias assessment

Supplement: Supplementary file 1 — Figure S1. Risk of bias assessment. [file BJO-125-944-s001.pdf]
