# Supplementary material for: Cervical dilatation patterns of ‘low‐risk’ women with spontaneous labour and normal perinatal outcomes: a systematic review
Source: BJOG. 2017 Nov 3;125(8):944–54. doi: 10.1111/1471-0528.14930 (PMC6033146; doi:10.1111/1471-0528.14930)
Supplement: Supplementary file 4 — Figure S4. Panel showing the distribution of time to gain 1 cm in parous women, by study. [file BJO-125-944-s004.pdf]

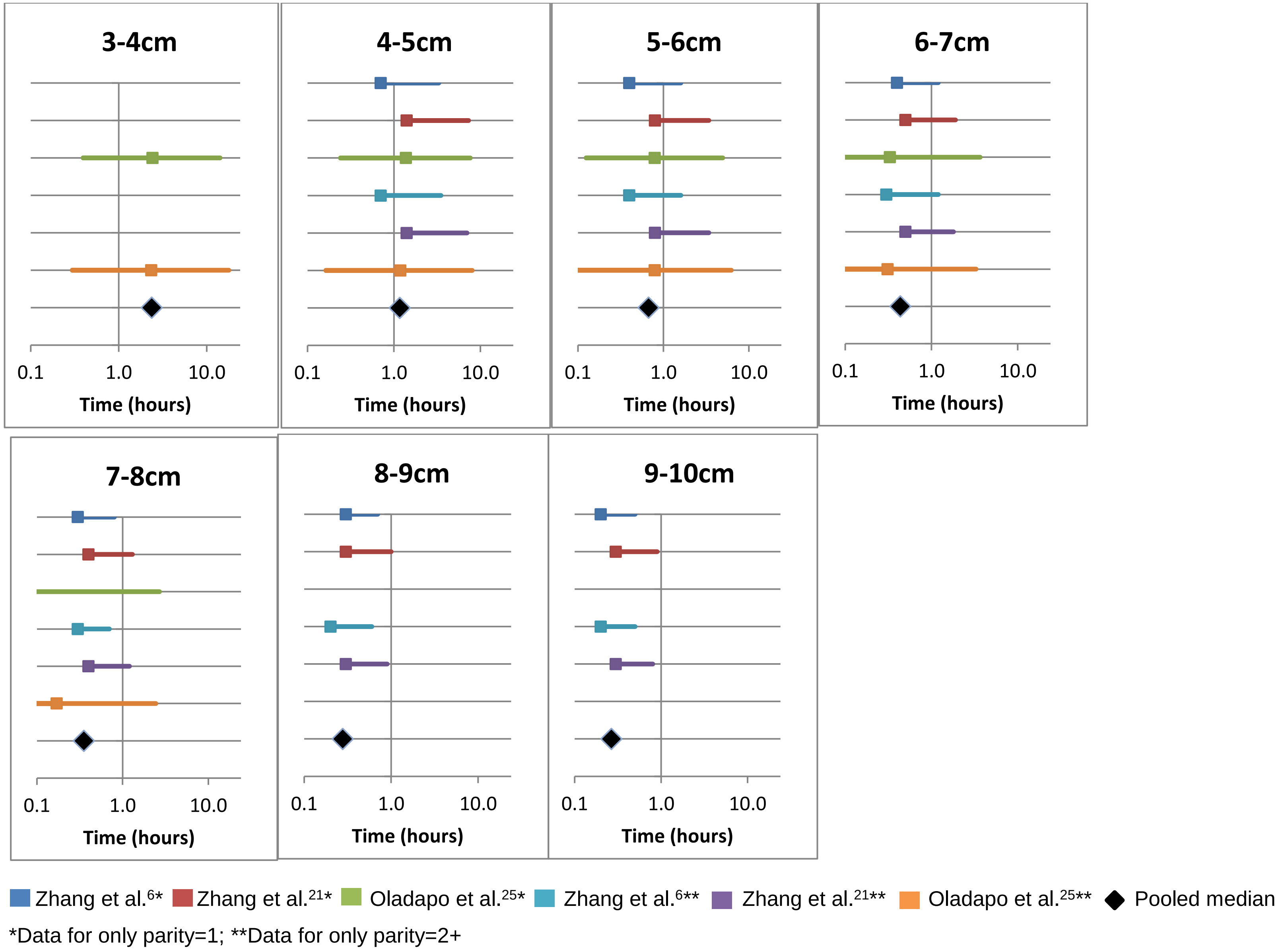

Figure S4. Panel showing the distribution of time to gain 1 cm in parous women by study
