# Supplementary material for: Cervical dilatation patterns of ‘low‐risk’ women with spontaneous labour and normal perinatal outcomes: a systematic review
Source: BJOG. 2017 Nov 3;125(8):944–54. doi: 10.1111/1471-0528.14930 (PMC6033146; doi:10.1111/1471-0528.14930)
Supplement: Supplementary file 6 — Figure S6. Panel showing 95th percentiles of cumulative duration of labour from admission, by study, among parous women. [file BJO-125-944-s006.pdf]

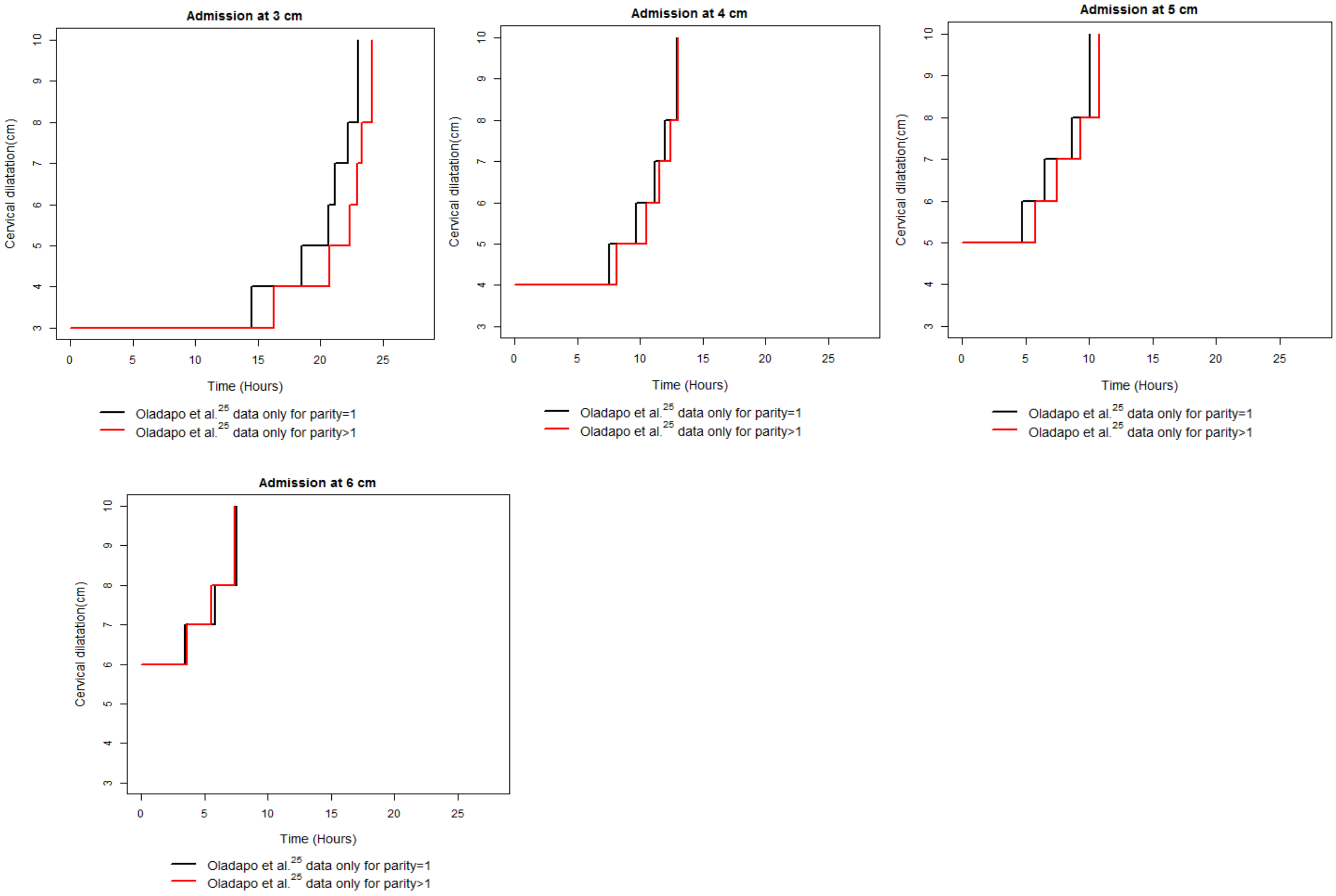

Figure S6. Panel showing 95th percentiles of cumulative duration of labour from admission by study among parous women
