# Supplementary material for: Cervical dilatation patterns of ‘low‐risk’ women with spontaneous labour and normal perinatal outcomes: a systematic review
Source: BJOG. 2017 Nov 3;125(8):944–54. doi: 10.1111/1471-0528.14930 (PMC6033146; doi:10.1111/1471-0528.14930)
Supplement: Supplementary file 7 — Table S1. Characteristics of included studies and study populations (nulliparous). [file BJO-125-944-s007.pdf]

Table S1. Characteristics of included studies and study populations (nulliparous)

Study	Year of publication	Study design	Years of data collection	Data collection method	Data analysis method	Setting	Sample size N=43148	Race/Ethnicity	Maternal age [mean, SD, years]	GA at delivery [mean (SD) weeks]	Maternal weight at delivery [mean (SD) kg]	BMI at delivery [mean (SD), kg/m²)	Birth weight [mean (range) g]
Chen et al.[23]	1986	Observational	1982-1983	Extraction of labour observations from labour chart	Details not described. Nomogram was constructed through linear regression.	Chan Gung Memorial Hospital, Taipei, Taiwan	500	Chinese:100%	24.3	39.5	_	_	3206 (2500-3850)
Zhang et al.[7]	2002	Observational	1992-1996	Extraction of labour observations from labour chart	Repeated-measures regression with a 10th-order polynomial function	Tripler Army Medical Centre, a tertiary care facility serving military personnel and their families in Hawaii, USA	1162	Non-Hispanic White: 65%; Non-Hispanic Black:12%; Hispanic: 7%; Asian: 11%; Other: 5%	23.0	39.3	76.7	_	3206 (2500-3850)
Suzuki et al.[24]	2010	Observational	2001-2005	Extraction of labour observations from labour chart	Repeated-measures regression with a 10th-order polynomial function	4 primary medical hospitals performing normal deliveries, Japan	2369	Japanese:100%	28.0(4.5)	39.8 (0.9)	61.7 (7.6)	_	3036 (2104-4210
Zhang et al.[6]	2010	Observational	1959-1965	Prospective data collection through in-person interviews, physical and laboratory exams and documentation in medical records	Repeated-measures regression with an 8th-order polynomial function	12 hospitals in the National Collaborative Perinatal Project, USA	8690	White: 53%; African- American: 39%; Other: 8%	20.3(3.9)	39.8 (1.4)	_	25.4 (3.5)	3185
Zhang et al.[21]	2010	Observational	2002-2008	Retrospective data extraction from electronic medical records	Repeated-measures regression with an 8th-order polynomial function	19 hospitals in the Consortium on Safe Labor Project, USA	27170	White: 60%; African- American: 12%; Hispanic: 20%; Asian or Pacific Islanders: 4%; Others: 4%	24.6(5.8)	39.3 (1.2)	_	29.1 (5.0)	3296
Shi et al.[22]	2016	Observational	2013-2014	Prospective data collection with the LaborPro system (Trig Medical Ltd, Yokneam, Israel)	Repeated-measures regression with a 8th-order polynomial function	First Affiliated Hospital of Chongqing Medical University, a university-based tertiary centre, China	1091	Chinese:100%	27.0(3.2)	39.5 (0.9)	_	26.1 (2.8)	3301
Oladapo et al.[25]	2017	Observational	2014-2015	Prospective data extraction following direct labour assessment	Survival analysis using interval censored times and Markov multistate model	13 secondary/tertiary level hospitals in Nigeria and Uganda	2166	Nigerian: 50.9%; Ugandan: 49.1%	25.1(4.2)	38.7(1.1)	71.8 (11.6)	28.1 (4.1)	3140
