# Supplementary material for: Cervical dilatation patterns of ‘low‐risk’ women with spontaneous labour and normal perinatal outcomes: a systematic review
Source: BJOG. 2017 Nov 3;125(8):944–54. doi: 10.1111/1471-0528.14930 (PMC6033146; doi:10.1111/1471-0528.14930)
Supplement: Supplementary file 8 — Table S2. Characteristics of included studies and study populations (parous). [file BJO-125-944-s008.pdf]

Table S2. Characteristics of included studies and study populations (parous)

Study	Year of publication	Study design	Years of data collection	Data collection method	Data analysis method	Setting	Sample size N=56823	Parity	Race/Ethnicity	Maternal age [mean, SD, years]	GA at delivery [mean (SD) weeks]	Maternal weight at delivery [mean (SD) kg]	BMI at delivery [mean (SD), kg/m²)	Birthweight (mean, g)
Zhang et al.*[6]	2010	Observational	1959-1965	Prospective data collection through in-person interviews, physical and laboratory exams and documentation in medical records	Repeated-measures regression with an 8th-order polynomial function	12 hospitals in the National Collaborative Perinatal Project, USA	6373	1	White: 55%; African- American: 38%; Other: 7%	22.7(4.4)	39.8 (1.4)	_	25.9	3259
Zhang et al.**[6]	2010	Observational	1959-1965	Prospective data collection through in-person interviews, physical and laboratory exams and documentation in medical records	Repeated-measures regression with an 8th-order polynomial function	12 hospitals in the National Collaborative Perinatal Project, USA	11765	>1	White: 45%; African- American: 48%; Other: 7%	27.4(5.5)	39.8 (1.4)	_	27.1	3301
Zhang et al.*[21]	2010	Observational	2002-2008	Retrospective data extraction from electronic medical records	Repeated-measures regression with an 8th-order polynomial function	19 hospitals in the Consortium on Safe Labour Project, USA	17850	1	White: 55%; African- American: 12%; Hispanic: 26%; Asian or Pacific Islanders: 4%; Others: 3%	27.7(5.7)	39.2 (1.2)	_	29.6	3384
Zhang et al.**[21]	2010	Observational	2002-2008	Retrospective data extraction from electronic medical records	Secondary analysis using repeated-measures regression with an 8th-order polynomial function	19 hospitals in the Consortium on Safe Labour Project, USA	17395	>1	White: 51%; African- American: 15%; Hispanic: 29%; Asian or Pacific Islanders: 2%; Others: 3%	30.0(5.4)	39.1 (1.1)	_	30.5	3384
Oladapo et al.*[25]	2017	Observational	2014-2015	Prospective data extraction following direct labour assessment	Survival analysis using interval censored times	13 secondary/tertiary level hospitals in Nigeria and Uganda	1488	1	Nigerian: 43.3%; Ugandan: 56.7%	27.1(4.1)	38.7 (1.1)	73.8	28.9	3278
Oladapo et al. **[25]	2017	Observational	2014-2015	Prospective data extraction following direct labour assessment	Nonlinear mixed model and survival analysis	13 secondary/tertiary level hospitals in Nigeria and Uganda	1952	>1	Nigerian: 40.6%; Ugandan: 59.4%	30.9(4.6)	38.8 (1.1)	76.4	29.7	3348

*Data only for parity=1, ** Data only for parity>1
