# Supplementary material for: Cervical dilatation patterns of ‘low‐risk’ women with spontaneous labour and normal perinatal outcomes: a systematic review
Source: BJOG. 2017 Nov 3;125(8):944–54. doi: 10.1111/1471-0528.14930 (PMC6033146; doi:10.1111/1471-0528.14930)
Supplement: Supplementary file 9 — Table S3. Risk of bias assessment of included studies. [file BJO-125-944-s009.pdf]

**Table S3.** Risk of bias assessment of included studies

| Study | Domain of assessment | Authors' judgment (Low/Unclear/High) | Support for judgment |
|---|---|---|---|
| **Chen et al.[23]** | Primary intent of the study research question | Low | The study was primarily designed to "define the progression of cervical dilatation of normal labour in Chinese primigravidae" (Pg. 573) |
| | Representativeness of the study population | Low | "500 primigravidas with normal labour" was included in the study. "Normal labour as defined as one in which patient was admitted in labor with cervical os dilatation of 3 cm or more and a Bishop score of 7 or more, had no interruption of labour of any kind such as induction, oxytocic agents, or lumbar epidural block, required no instrumental or abdominal delivery and gave birth to a baby appropriate for gestational age in good condition with body weight more than 2500 g." Parameters in Table 1 (Pg. 573) shows that all babies born were full term with mean Apgar score at 5 minutes of 9.6 and ranging between 7 and 10. |
| | Ascertainment and temporality of observations | Unclear | No description of how observations that were used to generate the time required for each cm of cervical dilatation. |
| | Adequacy of data points for valid assessment of cervical dilatation patterns | Unclear | No description of the number of vaginal examinations was provided. |
| | Use of a valid and robust approach for analysis and generation of labour curve | Unclear | No clear description of the approach used for determining the time required for each centimetre of dilatation. A straight line nomogram that was a derivative of "10 log (os dilatation) on the y-axis against mean time (h) for each cm os dilatation on the x-axis and by employing linear regression." Pg. 573. |
| | Primary intent of the | | We decided to re-examine the pattern |

| | | | |
|---|---|---|---|
| | study research question | Low | of labour progression among nulliparous women in contemporary practice using advanced statistical methods" (Pg. 824. " Because the purpose of our study was to demonstrate that a substantial proportion of labor ended in vaginal delivery may progress slower than the current cutoff points for labor arrest…" (Pg. 825). |
| **Zhang et al.**[7] | Representativeness of the study population | Unclear | " …nulliparous, singleton pregnancy, maternal age between 18 and 34 years, gestational age between 37 weeks 0 days and 41 weeks 6 days, birth weight between 2500 and 4000 g, spontaneous onset of labor, vertex presentation at admission, cervical dilation <7 cm at admission, and duration of labor from admission to delivery >3 hours…". There was no active management of labor or other special protocol. (Pg 825). However, newborn Apgar score were not reported. |
| | Ascertainment and temporality of observations | High | "We used data from a previous study in which detailed labor and delivery information was collected" (Pg. 824). In the cited study, data was retrospectively collected. "Labor and delivery nurses who went through special training for this study extracted detailed information from medical records to a standardized form" (Pg. 129 of the cited paper). |
| | Adequacy of data points for valid assessment of cervical dilatation patterns | Low | The median number of vaginal examinations in labor was 6 times (10th and 90th percentiles: 4 and 10 times, respectively) Pg. 826. |
| | Use of a valid and robust approach for analysis and generation of labour curve | Low | A repeated-measures regression with 10th order polynomial function was used to model the curve of cervical dilatation (Pg. 825). Statistical analysis was performed with SAS. |
| | Primary intent of the study research question | Low | "…the purpose of our research was to document the progress of nulliparous cervical dilation during normal delivery conditions and to create a cervical dilation model curve for comparison with both Friedman and Zhang curves". Pg. |

| | | | |
|---|---|---|---|
| | | | 226.e1. |
| **Suzuki et al.**[24] | Representativeness of the study population | Unclear | "Inclusion criteria were Japanese, singleton nulliparous pregnancy, gestational age between 37 weeks 0 days–41 weeks 6 days, spontaneous onset of labor, vertex presentation at admission, cervical dilation <7 cm at admission and duration of labor from admission to delivery <3 hours. We excluded those cases having cesarean delivery, labor induction, and epidural anesthesia" Pg. 226.e2. However, perinatal outcomes not explicitly reported although the aim of the study was reported as "… to document the progress of nulliparous cervical dilation during normal delivery conditions (Pg. 226.e1) |
| | Ascertainment and temporality of observations | High | "The study design was a retrospective review of medical records…" Pg. 226.e1. |
| | Adequacy of data points for valid assessment of cervical dilatation patterns | Low | 5 (2-16) in Table 1 on Pg. 226.e2. |
| | Use of a valid and robust approach for analysis and generation of labour curve | Low | A smoothing B-spline curve was created using the same approach as Zhang et al, which used a repeated-measures regression with 10th order polynomial function. Statistical analysis was performed with SAS. |
| | Primary intent of the study research question | Low | "The objective of our study was to examine labor patterns in a large population and to explore an alternative approach for diagnosing abnormal labor progression" (Pg. 705). |
| **Zhang et al.**[6] | Representativeness of the study population | Low | "We restricted our analysis to term pregnancies (37–42 weeks), with a spontaneous onset of labor. We also restricted to pregnancies with vertex fetal presentation at admission (excluding face, brow, or chin position. To examine the labor pattern in parturients with normal perinatal outcomes, we restricted our analysis to births of neonates with 5-minute Apgar scores of at least 7 neonates with 5-minute.  Further excluded were cases of placenta praevia, severe hypertension in pregnancy, cord prolapse, and uterine rupture. Women |

| | | | |
|---|---|---|---|
| | | | who had fewer than two values of cervical dilation in the first stage or never reached 10 cm (Pg. 706)." |
| | Ascertainment and temporality of observations | Low | "…We sought to study the first stage of labor using data from the National Collaborative Perinatal Project, a large, multicenter, prospective, observational study conducted between 1959 and 1966."(Pg. 705). |
| | Adequacy of data points for valid assessment of cervical dilatation patterns | Low | "Total number of pelvic exams in first stage was 6 (3, 11), 5 (2, 9), 5 (2, 9) for P0, P1, and P2+, respectively" in Table 1 (Pg. 707). |
| | Use of a valid and robust approach for analysis and generation of labour curve | Low | Repeated-measures regression with 8th order polynomial model was used to construct average labour curves by parity. Labour progression data were interval censored. Statistical analysis was performed with SAS 9.1 (Pg. 707). |
| **Zhang et al.** [21] | Primary intent of the study research question | Low | "The purpose of this study was to use contemporary labor data in a large number of parturients with spontaneous onset of labor to examine the labour patterns and estimate the duration of labor in the United States". Pg. 1282. |
| | Representativeness of the study population | Low | Diagram of patient selection in Fig 1. Pg. 1282. |
| | Ascertainment and temporality of observations | Low | "… a multicenter retrospective observational study that abstracted detailed labor and delivery information from electronic medical records in 12 clinical centers…" Pg. 1282 |
| | Adequacy of data points for valid assessment of cervical dilatation patterns | Low | Total number of vaginal exams in first stage (median, 10th, 90th percentiles) Parity 0: 5 (1, 9) Parity 1: 4 (1, 7) Parity+2: 4 (1, 7) |
| | Use of a valid and robust approach for analysis and generation of labour curve | Low | " We used a repeated-measures analysis with 8th degree polynomial model to construct average labor curves by parity" |
| **Shi et al.**[22] | Primary intent of the study research question | Low | "The aim of our study was to generate evidence to re-evaluate labour patterns in Chinese women" (Pg. 58) |
| | Representativeness of the study population | Low | "We selected Chinese women with a singleton pregnancy with a gestational age between 37 weeks 0 days and 41 weeks 6 days, vertex presentation at admission and spontaneous onset of labour. Women were excluded if there |

| | | | |
|---|---|---|---|
| | | | was evidence of obesity [body mass index (BMI) ≥30 kg/m2] before pregnancy, use of analgesia in labour, trial of labour after previous CS or other medical conditions…". Participants with any adverse perinatal outcomes, such as fetal distress, Apgar score ≤7 at 5 minutes, resuscitation and/or admission to the neonatal intensive care unit, birth injury, stillbirth, infant death, postpartum haemorrhage, puerperal infection, serious laceration of the soft birth channel or maternal death during the 6-week follow up, were also excluded from the analysis (Pg. 58) |
| | Ascertainment and temporality of observations | Low | "The data were prospectively collected between 1 September 2013 and 31 August 2014 from women at the First Affiliated Hospital of Chongqing Medical University…(Pg. 59)". |
| | Adequacy of data points for valid assessment of cervical dilatation patterns | Low | Table 1 |
| | Use of a valid and robust approach for analysis and generation of labour curve | Low | A repeated-measures regression with 10th order polynomial function was used to model the curve of cervical dilatation (Pg. 59). Statistical analysis was performed with SAS. |
| | Primary intent of the study research question | Low | "..We examined the patterns of labour progression in a prospective cohort of African women with normal perinatal outcomes following spontaneous labour onset and vaginal birth." |
| **Oladapo et al.[25]** | Representativeness of the study population | Low | "...we restricted our analysis to examine labour patterns and curves to 5606 women on the basis of the following inclusion criteria (Figure 1): term births (between 37 weeks and 0 days and 41 weeks and 6 days) with vertex presentation and spontaneous labour onset. We excluded women who had labour induction, previous uterine scar, or caesarean section (during labour). To examine the labour patterns in women with normal perinatal outcomes, we excluded women whose labour resulted in severe adverse outcomes which was defined as occurrence of any of the |

| | | |
|---|---|---|
| | | following: stillbirths, early neonatal deaths, neonatal use of anticonvulsants, neonatal cardio-pulmonary resuscitation, Apgar score < 6 at 5 minutes, maternal death or organ dysfunction with labour dystocia and uterine rupture". |
| Ascertainment and temporality of observations | Low | "From the medical record, trained research nurses prospectively extracted detailed information…" |
| Adequacy of data points for valid assessment of cervical dilatation patterns | Unclear | Total number of vaginal exams in first stage (median, 10th, 90th percentiles) Parity 0: 3(2, 5) Parity 1: 3 (2, 4) Parity+2: 3 (2, 4) |
| Use of a valid and robust approach for analysis and generation of labour curve | Low | " We independently applied survival analysis and multistate Markov models to estimate the duration of labour centimetre by centimetre until 10 cm; and the cumulative duration of labour from dilatation at admission through to 10 cm. Nonlinear mixed models and multistate Markov models were separately used to construct average labour curves. All analyses were conducted according to three parity groups: parity=0 (n=2166), parity=1 (n=1488), and parity=2+ (n=1952). We performed sensitivity analyses to assess the impact of oxytocin augmentation on labour progression by reexamining the progression patterns after excluding women with augmented labours." |
