# Supplementary material for: Cervical dilatation patterns of ‘low‐risk’ women with spontaneous labour and normal perinatal outcomes: a systematic review
Source: BJOG. 2017 Nov 3;125(8):944–54. doi: 10.1111/1471-0528.14930 (PMC6033146; doi:10.1111/1471-0528.14930)
Supplement: Supplementary file 10 — Table S4. Overall risk of bias assessment. [file BJO-125-944-s010.pdf]

Table S4. Overall risk of bias assessment

Risk of bias domain	Chen et al.[23]	Zhang et al.[7]	Suzuki et al.[24]	Zhang et al.[6]	Zhang et al.[21]	Shi et al.[22]	Oladapo et al.[25]
Primary intent of the study research question	+	+	+	+	+	+	+
Representativeness of the study population	+	?	?	+	+	+	+
Ascertainment and temporality of observations	?	--	--	+	+	+	+
Adequacy of data points for valid assessment	?	+	+	+	+	+	--
Use of a valid and robust approach for analysis	?	+	+	+	+	+	+
Overall risk of bias	++	+++	+++	+++++	+++++	+++++	++++

+ Low risk of bias, ? Unclear risk of bias, -- High risk of bias
