# Supplementary material for: Cervical dilatation patterns of ‘low‐risk’ women with spontaneous labour and normal perinatal outcomes: a systematic review
Source: BJOG. 2017 Nov 3;125(8):944–54. doi: 10.1111/1471-0528.14930 (PMC6033146; doi:10.1111/1471-0528.14930)
Supplement: Supplementary file 13 — Appendix S1. Search strategies (date of search: 15 December 2016). [file BJO-125-944-s013.pdf]

Appendix S1. Search strategies (date of search: 15 December 2016)

#	Database : PubMed Interface : http://www.pubmed.gov	Results
1	"labor curve" [TW] OR "labour curve" [TW] OR "labor curves" [TW] OR "labour curves" [TW]	69
2	(Cervical [TW] OR cervix [TW] OR "cervix uteri" [MH]) AND (Dilation [TW] OR Dilatating [TW] OR Dilatated [TW] OR Dilatation [TW] OR dilated[TW] OR dilating [TW] OR "DILATATION" [MH] OR "Dilatation, Pathologic"[Mesh])	5467
3	"Labor, Obstetric"[Mesh] OR delivery, obstetric[MeSH Terms] OR labor [TW] OR labour [TW] OR "obstetric delivery" OR "obstetric deliveries" OR obstetric*[TIAB] OR "Parturition"[Mesh] OR birth* [TW] OR childbirth* [TW] OR parturition* [TW]	511935
4	Step 2 AND Step 3	2797
5	Step 1 OR Step 4	2834
**#**	**Database EMBASE : Interface : http://www.embase.com**	**Results**
1	((cervical OR cervix) NEAR/2 (dilat* OR dilatating OR dilatated OR dilated OR dilating OR dilation)):ti,ab,de	5728
2	'uterine cervix'/exp AND 'dilatation'/exp	29
3	'obstetrics'/exp OR labor:ti,ab,de OR labour:ti,ab,de OR childbirth:ti,ab,de OR birth:ti,ab,de OR 'childbirth'/exp OR partus:ti,ab,de OR parurition:ti,ab,de OR 'obstetric procedure'/exp	821001
4	(Step 1 OR Step 2) AND Step 3	5315
5	'labor curve':ti,ab,de OR 'labour curve':ti,ab,de OR 'labor curves':ti,ab,de OR 'labour curves':ti,ab,de OR 'uterine cervix dilatation'/exp	3845
6	Step 5 OR Step 4	5366
7	/lim to EMBASE	4684
**#**	**Database : POPLINE Interface : http://www.popline.org**	**Results**
1	(((Keyword:CERVICAL DILATATION))) OR (((Title:((Cervical OR Title:cervix)) AND (Title:(Dilat* OR Title:dilated OR Title:dilating))))) OR (((Title:"labor curve" OR Title:"labour curve" OR Title:"labor curves" OR Title:"labour curves")))	424
**#**	**Database : Global Index Medicus Interface : http://pesquisa.bvsalud.org/**	**Results**
1	((tw:(labor OR labour)) AND (tw:(curve* OR curve*))) OR ((tw:(cervical OR cervic)) AND (tw:(dilat*)))	581
**#**	**Database CINAHL Full Text: Interface : http**	**Results**
1	((AB cervical OR AB cervix OR AB "uterine cervix " OR MH cervix) AND (AB Dilat* OR AB dilated OR AB dilating OR MH dilatation OR AB ripening)) OR MH "cervix dilatation and effacement" OR AB "labor curve" OR AB "labour curve" OR AB "labor curves" OR AB "labour curves"	
2	((TI cervical OR TI cervix OR TI "uterine cervix " OR MH cervix) AND (TI Dilat* OR TI dilated OR TI dilating OR MH dilatation OR TI ripening)) OR MH "cervix dilatation and effacement" OR TI "labor curve" OR TI "labour curve" OR TI "labor curves" OR TI "labour curves"	
3	Step 1 OR Step 2	1211
4	Exclude Medline Records	262
